# Nitric Oxide-Based Signaling During Abiotic Stress Responses in Plants: Mechanisms of Tolerance and Applicability in Sustainable Horticultural Crop Management

**DOI:** 10.3390/plants15050825

**Published:** 2026-03-07

**Authors:** Tiba Nazar Ibrahim Al Azzawi, Murtaza Khan, Yong Ha Rhie

**Affiliations:** 1 Department of Horticulture, Kangwon National University, Chuncheon 24341, Republic of Korea; redflower660@yahoo.com; 2 Agriculture and Life Science Research Institute, Kangwon National University, Chuncheon 24341, Republic of Korea; murtazakhan@kangwon.ac.kr; 3 Interdisciplinary Program in Smart Agriculture, Kangwon National University, Chuncheon 24341, Republic of Korea

**Keywords:** nitric oxide signaling, abiotic stress tolerance, redox regulation, horticultural crops, sustainable stress management

## Abstract

Abiotic stresses severely constrain the growth, yield, and quality of horticultural plants, collectively posing major challenges to sustainable production under changing climatic conditions. Nitric oxide (NO) is a key signaling molecule that modulates plant responses to abiotic stress by integrating with redox regulation systems, hormonal crosstalk pathways, ion homeostasis mechanisms, and transcriptional control networks. Rather than functioning as an isolated regulator, NO participates in dynamic signaling frameworks whose outcomes depend on concentration, timing, cellular redox status, and interaction with other signaling molecules. This review synthesizes current knowledge on NO-mediated mechanisms contributing to abiotic stress tolerance and examines their relevance to sustainable horticultural crop management. After outlining the historical recognition of NO as a plant signaling molecule, we discuss stress-responsive NO-dependent processes, including S-nitrosylation-based post-translational modification, NO–reactive oxygen species (ROS) interactions, and the modulation of stress-responsive transcriptional programs. The roles of NO in tolerance to drought, salinity, extreme temperature, and heavy metal stress are analyzed with emphasis on experimentally supported physiological and molecular responses. We further evaluate evidence from fruit, vegetable, ornamental, and medicinal crops, highlighting how NO-associated signaling correlates with yield stability, quality-related traits, and post-harvest performance under stress conditions. Finally, NO-based strategies such as priming, donor application, and integration with biostimulants are critically assessed in the context of climate-resilient and sustainable horticulture, with attention to translational constraints and field-level feasibility. By connecting mechanistic insights with applied considerations, this review provides a structured framework for evaluating the potential and limitations of NO-based approaches in abiotic stress management of horticultural crops.

## 1. Introduction

Plants are continuously exposed to a broad range of abiotic stresses, including drought, salinity, extreme temperatures, and heavy-metal (HM)-induced toxicity, collectively posing a major threat to global agricultural productivity and food security. These stresses disrupt cellular homeostasis, impair metabolic processes, and reduce crop yield and quality. Such impacts are particularly pronounced in horticulture crops, whose productivity, post-harvest performance, and market value are highly sensitive to environmental fluctuations [1,2]. Climate change is intensifying the frequency and severity of abiotic stresses, necessitating a deeper mechanistic understanding of plant stress responses and the development of sustainable management strategies.

Abiotic stress responses are governed by complex signaling networks that integrate environmental cues with endogenous regulatory systems. Among the signaling molecules implicated in these processes, nitric oxide (NO) has emerged as an important modulator. NO, a bioactive molecule, was initially identified in studies of animal physiology as an endothelium-derived relaxing factor and a signaling molecule. Subsequent research established its presence and signaling functions in plants, where it contributes to the regulation of growth, development, and responses to both biotic and abiotic stimuli [3,4]. Unlike classical phytohormones, NO is a low-molecular-weight, highly reactive gaseous molecule with rapid diffusion and dynamic regulatory capacity, enabling it to function as a versatile signal under stress conditions.

Accumulating evidence indicates that NO contributes to abiotic stress tolerance by modulating redox homeostasis, antioxidant defense systems, ion transport processes, osmotic adjustment, and the expression of stress-responsive genes [5,6]. A central mechanism by which NO exerts regulatory influence is redox-based post-translational modification, particularly S-nitrosylation, which alters the activity, stability, and localization of target proteins involved in stress adaptation [7]. Importantly, NO does not operate independently of other redox signals. Instead, NO-reactive oxygen species (ROS) interactions form a dynamic signaling interface that shapes cellular responses depending on relative concentrations, spatial distribution, and the efficiency of antioxidant and denitrosylation systems [5,8]. This balance determines whether NO contributes to adaptive signaling or to nitrosative stress.

In addition to redox interactions, NO participates in signaling crosstalk with major phytohormones, including abscisic acid (ABA), auxins (AUX), salicylic acid (SA), ethylene (ETH), and jasmonates (JAs) [9,10,11]. Through post-translational modification of signaling components, modulation of transcription factor activity, and interaction with secondary messengers, NO influences processes such as stomatal regulation, root system architecture, photosynthetic efficiency, and stress memory formation. These effects are context-dependent and vary with species, developmental stage, stress intensity, and cellular redox status.

In recent years, approaches involving the use of NO donors and priming, as well as integration with biostimulants to enhance stress resilience in crops, have gained increasing attention [12,13]. In horticultural systems, experimental studies have associated NO-based treatments with improved stress tolerance, yield stability, fruit quality, and post-harvest shelf life under adverse environmental conditions [14]. However, most evidence derives from controlled-environment studies, and the scalability and consistency of such interventions under field conditions remain areas requiring further investigation.

Despite significant progress in elucidating NO-related signaling mechanisms, major challenges remain in translating laboratory-based insights into field-level applications [15]. Responses to NO are highly dependent on concentration, exposure duration, tissue type, and the pre-existing redox environment. Moreover, excessive or prolonged exposure to NO may disrupt redox balance and induce nitrosative stress, underscoring the importance of precise regulation [16]. Crop-specific variability further complicates the design of universal NO-based management strategies.

This review synthesizes current knowledge on NO-associated mechanisms contributing to plant tolerance to abiotic stress, with particular emphasis on their implications for sustainable horticultural crop management. Rather than presenting NO as an isolated or dominant regulator, we examine its role within broader signaling networks, highlighting mechanistic evidence, stress-specific responses, and translational considerations. By integrating molecular insights with crop-level perspectives, this review aims to provide a balanced and evidence-based framework for evaluating the potential of NO-centered strategies in sustainable horticulture.

## 2. A Brief Historical Perspective on NO in Plant Stress Biology, Literature Selection, and Scope of the Review

NO, as a bioactive molecule, was initially recognized during studies of animal physiology, where it was identified as an endothelium-derived relaxing factor and a signaling molecule. Its relevance in plant biology began to gain attention in the late 1990s, when seminal studies demonstrated that NO participates in defense responses and stress signaling rather than merely representing a toxic byproduct of metabolism. One of the earliest breakthroughs was the observation of rapid NO accumulation in response to environmental stimuli and pathogen challenge, suggesting a signaling function analogous, in principle, to that described in animal systems [17].

Subsequent investigations extended this concept to abiotic stress biology, demonstrating that NO is involved in plant responses to drought, salinity, and oxidative stress. Early physiological investigations revealed that exogenous application of NO donors can alleviate stress-induced damage by enhancing antioxidant capacity, stabilizing cellular membranes, and improving photosynthetic performance. These findings shifted the perception of NO from a stress-induced reactive molecule to a regulatory signaling molecule involved in stress tolerance [18,19]. Importantly, these studies did not imply that NO acts independently; rather, they indicated that NO modulates pre-existing redox and stress-response pathways.

During the 2000s, advances in redox biology and molecular techniques strengthened the mechanistic understanding of NO function in plants. The discovery of S-nitrosylation as a reversible post-translational modification provided a biochemical basis for NO-mediated regulation of protein function under stress conditions. This modification was shown to alter enzyme activity, protein–protein interactions, and subcellular localization, thereby influencing stress signaling outputs. In parallel, research highlighted extensive NO–ROS interactions, reinforcing the concept that NO operates within a broader redox signaling framework rather than in isolation [8,20]. The recognition that the balance between ROS production, antioxidant defenses, and denitrosylation systems determines the physiological outcome represented a major conceptual advance in plant stress biology.

More recently, attention has shifted toward elucidating how NO integrates with hormone-based signaling pathways and transcriptional regulation processes. Studies have demonstrated that NO can modulate components of ABA, JAs, SA, and ETH signaling cascades through redox-dependent mechanisms, thereby influencing stress-responsive gene expression patterns [5,10,21]. These developments have positioned NO not as a standalone regulator but as a context-dependent modulator embedded within complex signaling networks.

Building upon these milestones, the present review focuses on experimentally supported mechanisms through which NO contributes to abiotic stress tolerance and examines how such knowledge may inform sustainable management strategies in horticultural crops. The historical trajectory of NO research thus provides a conceptual foundation for evaluating both its mechanistic roles and its translational potential.

This review synthesizes current knowledge on NO-mediated abiotic stress tolerance in plants, with emphasis on horticultural crops and translational relevance. Peer-reviewed articles were identified using major scientific databases, including Web of Science, Scopus, and PubMed, employing combinations of keywords such as “nitric oxide,” “abiotic stress,” “drought,” “salinity,” “temperature stress,” “heavy metals,” “horticultural crops,” “priming,” and “NO donors.”

Priority was given to experimental studies published within the last 15 years, while foundational earlier research was included to provide mechanistic context. Both laboratory- and greenhouse-based studies were considered, along with available field investigations. Given the heterogeneity of experimental systems, donor types, stress intensities, and crop species, the present work adopts a structured critical synthesis approach rather than a quantitative meta-analysis.

Where possible, evidence was comparatively evaluated across stress types and crop categories to identify consistent mechanistic patterns, translational gaps, and limitations. Particular attention was given to distinguishing mechanistic insights derived from controlled environments from data supported by field validation.

## 3. Stress-Responsive NO-Based Signaling Framework in Plants

NO-based signaling in plants is highly dynamic and context-dependent, with its production, localization, and functional consequences strongly influenced by abiotic stress conditions. Rather than acting through a single linear pathway, NO participates in interconnected signaling networks that respond rapidly to environmental perturbations, such as drought, salinity, temperature extremes, and HM exposure. Under these conditions, transient changes in NO concentration function as modulatory signals that contribute to the activation of protective responses while maintaining cell redox balance [22].

Abiotic stress conditions often trigger localized and temporally regulated accumulation of NO in coordination with alterations in cellular redox status. Stress-induced NO generation is closely associated with fluctuations in ROS levels, forming an integrated redox signaling network that determines whether cells undergo oxidative damage or adaptive acclimation. Moderate and tightly controlled NO production promotes stress tolerance by activating antioxidant enzymes, stabilizing membranes, and modulating ion transport processes. In contrast, excessive or prolonged NO accumulation may exacerbate nitrosative stress, leading to protein dysfunction and cellular injury [8,20,23]. Thus, the physiological outcome depends on quantitative and spatial parameters rather than the mere presence of NO.

A defining feature of stress-responsive NO signaling is the regulation of protein function through redox-based post-translational modifications, particularly S-nitrosylation. This reversible modification enables rapid control of key enzymes, transcription factors (TFs), and signaling components involved in stress perception and downstream responses. Under abiotic stress, S-nitrosylation has been reported to affect antioxidant enzymes, hormone signaling intermediates, and proteins associated with stomatal regulation, thereby contributing to multi-level physiological adjustment [7,24]. The reversibility of S-nitrosylation, mediated by denitrosylating systems such as thioredoxins and S-nitrosoglutathione reductase (GSNOR), further underscores the dynamic nature of NO-dependent regulation.

Spatial compartmentalization adds another layer of specificity to NO signaling during stress. NO production and action within chloroplasts, mitochondria, peroxisomes, and the apoplast allow plants to tailor responses to the metabolic processes most affected by stress. For example, chloroplast-localized NO can influence photosynthetic performance and ROS detoxification, whereas mitochondrial NO may modulate respiratory metabolism and redox homeostasis. Temporal dynamics are equally important: early NO bursts often function as triggers for downstream signaling cascades, whereas sustained or oscillatory NO patterns may participate in longer-term acclimation processes [25].

Crucially, NO-based signaling intersects extensively with phytohormone pathways, including ABA, ETH, JA, and SA. Through redox-dependent modulation of signaling components and transcriptional regulators, NO can influence stomatal closure, root system remodeling, and stress-responsive gene expression. These interactions do not imply hierarchical control but rather reflect bidirectional crosstalk within a broader signaling network [5,20,26,27]. Collectively, these features define a stress-responsive NO signaling framework in which concentration, localization, timing, and interaction with ROS and hormonal pathways determine physiological outcomes. This network perspective provides a mechanistic foundation for evaluating NO-centered strategies in sustainable abiotic stress management.

## 4. Molecular Mechanisms of NO-Mediated Abiotic Stress Tolerance

NO contributes to abiotic stress tolerance through a suite of interconnected molecular mechanisms that influence stress perception, redox homeostasis, and adaptive reprogramming. Rather than functioning as an isolated messenger, NO operates within integrated signaling networks involving redox regulation, ROS dynamics, phytohormone pathways, and transcriptional control systems. The outcome of NO signaling depends on its concentration, subcellular localization, timing, and interaction with other signaling molecules. These parameters collectively determine whether plants undergo stress-induced injury or achieve physiological acclimation.

### 4.1. Redox Signaling and S-Nitrosylation

Redox-based signaling represents a central mechanism through which NO modulates plant responses to abiotic stress. One of the most crucial NO-dependent post-translational modifications is S-nitrosylation, a reversible covalent modification of cysteine residues that can alter protein conformation, catalytic activity, stability, and protein–protein interactions. Under abiotic stress, S-nitrosylation enables rapid and reversible regulation of proteins involved in antioxidant defense, metabolic adjustment, and stress-signaling cascades [7].

Several key antioxidant enzymes, including ascorbate peroxidase, catalase, and superoxide dismutase, are subject to S-nitrosylation. Depending on the redox context and modification site, S-nitrosylation may either enhance or transiently suppress enzyme activity, thereby fine-tuning ROS scavenging capacity in a stress-dependent manner. This reversible modulation helps prevent excessive oxidative damage while maintaining ROS at signaling-competent levels necessary for downstream responses [8,28].

Beyond antioxidant systems, S-nitrosylation affects metabolic enzymes and ion transporters implicated in osmotic and ionic balance under drought and salinity stress. For example, modification of membrane-associated proteins can influence ion fluxes, while changes in metabolic enzyme activity can redirect carbon and energy metabolism toward stress adaptation. Such effects highlight the versatility of NO-mediated redox control as a modulatory, rather than purely stimulatory, mechanism.

Importantly, the specificity and reversibility of S-nitrosylation are dictated by the cellular redox status, local NO concentrations, cysteine accessibility, and the presence or absence of denitrosylating systems such as GSNOR and thioredoxins [29]. These systems remove nitrosyl groups and restore protein function, thereby preventing excessive accumulation of S-nitrosylated proteins under prolonged or severe stress. Consequently, NO-dependent redox signaling is best understood as a dynamic equilibrium between nitrosylation and denitrosylation processes that collectively determine physiological outcomes.

### 4.2. Interplay Between NO and ROS

NO–ROS interaction constitutes a core regulatory module in plant abiotic stress responses. Abiotic stresses frequently induce ROS accumulation, which can function as both damaging oxidants and essential secondary messengers. NO modulates this dual role by influencing ROS production, scavenging, and downstream signaling pathways [8,30].

At moderate levels, coordinated NO and ROS production can activate defense-associated signaling cascades, including MAPK pathways and transcriptional regulators. NO influences ROS homeostasis by modulating antioxidant enzyme activity and affecting electron transport processes in chloroplasts and mitochondria, where major ROS generation occurs [31]. Conversely, ROS can also alter NO bioavailability by affecting nitric oxide synthase-like activity, nitrate reductase pathways, and redox-sensitive NO storage forms, thereby shaping signaling efficiency [32].

The formation of reactive nitrogen species (RNS), such as peroxynitrite, further illustrates the biochemical mechanisms of NO and ROS pathways. Peroxynitrite can nitrate tyrosine residues and modify protein function, adding another layer of post-translational regulation [33]. Whether these reactions promote adaptation or contribute to cellular damage depends on concentration thresholds, subcellular localization, and antioxidant buffering capacity.

During stress acclimation, transient and spatially restricted bursts of NO and ROS often function as early warning signals that initiate protective gene expression and metabolic adjustments. In contrast, sustained imbalance between ROS generation and NO-mediated modulation may lead to oxidative or nitrosative injury [34]. Thus, stress tolerance is associated not with elevated NO or ROS per se, but with coordinated redox homeostasis.

### 4.3. Hormone Crosstalk During NO-Mediated Stress Responses

NO-mediated signaling intersects extensively with phytohormone pathways, allowing integration of stress adaptation with growth regulation. Among these interactions, NO–ABA crosstalk plays a central role in tolerance to drought and salinity. In guard cells, NO acts downstream of ABA signaling and contributes to calcium-dependent signaling events and ion channel modulation that promote stomatal closure under water deficit conditions [27,35,36]. S-nitrosylation of signaling components within the ABA pathway has been proposed as one mechanism underlying this modulation.

NO also interacts with AUX signaling to modulate root system architecture. By modulating auxin transporters and signaling intermediates through redox-dependent mechanisms, NO can affect root elongation and lateral root formation, thereby enhancing soil exploration under drought or salinity stress. These effects are context-dependent and influenced by hormone concentration gradients and redox status.

Crosstalk between NO and ETH, JA, and SA further contributes to stress-responsive regulation of senescence, antioxidant defenses, and defense-related gene expression [37,38]. These interactions do not indicate hierarchical dominance of NO but instead reflect bidirectional signaling relationships in which NO both influences and is influenced by hormonal pathways.

Through these hormone interactions, NO participates in integrating environmental stress cues with endogenous developmental programs, facilitating adaptive reprogramming rather than generalized growth inhibition [39].

### 4.4. Regulation of Stress-Responsive Genes and Transcription Networks

At the transcription level, NO contributes to abiotic stress tolerance by modulating stress-responsive gene expression. This regulation may occur directly through redox-based modifications of TFs or indirectly via ROS- and hormone-mediated signaling cascades. Stress-responsive TF families, such as bZIP, NAC, MYB, and WRKY, are sensitive to NO-mediated regulation and control genes associated with antioxidant defense, osmoprotection, detoxification, and metabolic adjustment [40,41].

NO has been implicated in chromatin remodeling and epigenetic regulation under adverse conditions, suggesting a potential role in stress memory and long-term acclimation [42]. Although the precise mechanisms remain under investigation, redox-sensitive regulation of histone-modifying enzymes and chromatin-associated proteins provides a plausible mechanistic link.

By coordinating transcription reprogramming with redox and hormone-based signaling, NO contributes to rapid yet flexible adaptive responses to abiotic stress [43]. However, these effects depend on quantitative and contextual parameters, including stress severity and cellular redox buffering capacity.

Collectively, these interconnected mechanisms illustrate how NO participates in linking stress perception with physiological and developmental adjustment. Rather than functioning as a solitary regulator, NO acts as a context-dependent modulator embedded within broader redox and hormonal signaling networks. Understanding these integrated pathways provides a mechanistic basis for evaluating the potential—and limitations—of NO-centered approaches in sustainable crop stress management strategies [44].

## 5. NO in Major Abiotic Stress Tolerance Mechanisms

NO has been widely implicated in plant responses to diverse abiotic stresses; however, its function is neither isolated nor universally beneficial. Rather, evidence from controlled experimental systems indicates that NO operates within integrated redox and hormonal signaling networks, where its effects depend on concentration, spatial distribution, stress intensity, developmental stage, and the prevailing cellular redox status [45]. While core signaling principles—such as S-nitrosylation, ROS modulation, and hormone crosstalk—are shared across stress types, downstream molecular targets and physiological outcomes are stress-specific and context-dependent [46].

**Figure 1:**
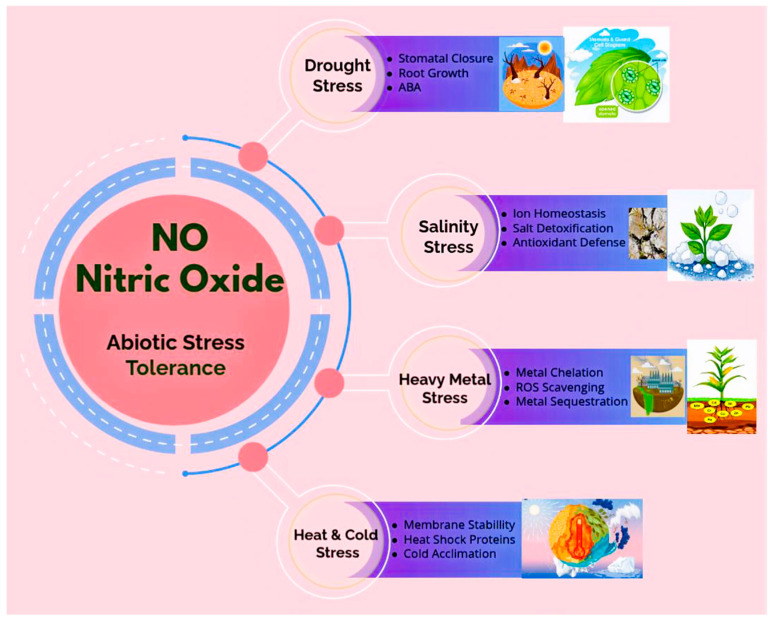
Conceptual model summarizing nitric oxide (NO)-associated signaling modules involved in plant responses to major abiotic stresses.

Figure 1 summarizes current evidence regarding NO-associated signaling modules involved in major abiotic stresses. The model should be interpreted as a conceptual framework derived primarily from laboratory and greenhouse studies, rather than as proof of a universally dominant regulatory role for NO.

The figure illustrates stress-specific signaling components reported in the literature for drought, salinity, temperature extremes, and heavy metal exposure. NO-associated processes include modulation of stomatal regulation, ion homeostasis, antioxidant defense, membrane stability, metal chelation, and stress-responsive gene expression. The model represents a schematic integration of findings primarily derived from controlled laboratory and greenhouse studies and should be interpreted as a conceptual framework rather than evidence of a universally dominant regulatory role for NO. The magnitude and outcome of NO-mediated effects depend on concentration, cellular localization, stress intensity, developmental stage, and interaction with ROS and hormonal signaling networks, as discussed in Section 5.1, Section 5.2, Section 5.3 and Section 5.4.

### 5.1. Drought

Drought stress triggers transient increases in endogenous NO levels in roots and guard cells in several species, including horticultural crops. These NO fluctuations often occur downstream of ABA signaling and contribute to stomatal regulation under water deficit [47].

Mechanistically, NO participates in guard cell signaling by modulating Ca^2+^ influx, activating anion channels, and influencing K^+^ efflux pathways. In some systems, S-nitrosylation of signaling proteins and interaction with ROS-producing NADPH oxidases have been reported, suggesting that stomatal closure results from coordinated NO–ROS signaling rather than NO alone [48,49]. The magnitude and duration of NO accumulation appear critical; moderate, transient NO production is associated with adaptive stomatal control, whereas sustained accumulation may contribute to nitrosative stress.

Beyond stomatal responses, drought-induced NO has been linked to modulation of antioxidant enzyme activity, partly through reversible S-nitrosylation. This regulation contributes to maintaining ROS at signaling-competent levels while limiting oxidative injury [50,51,52,53]. In roots, NO has been reported to influence architecture remodeling, possibly via interaction with auxin transport and signaling pathways, thereby enhancing soil water foraging under moderate stress [54,55]. Importantly, these effects vary across species and experimental conditions, indicating that NO-mediated drought tolerance is highly context-dependent rather than universal.

### 5.2. Salinity

Salinity imposes both osmotic stress and ionic toxicity, primarily due to excessive Na^+^ accumulation. Under such conditions, altered NO production has been documented in multiple plant systems [56,57]. Available evidence suggests that NO can influence ion homeostasis by modulating transporter activity, including Na^+^ extrusion and K^+^ retention mechanisms, although the precise molecular targets differ among species [58,59,60].

Crucially, salinity-induced responses frequently involve coordinated NO–ROS dynamics. NO may regulate antioxidant gene expression and enzyme activity, while ROS can reciprocally influence NO bioavailability, forming a tightly coupled redox signaling module [6,61]. The balance between these signals determines whether cells undergo adaptive acclimation or oxidative/nitrosative damage.

NO has also been associated with ABA-dependent osmotic adjustment and stress-responsive transcriptional reprogramming. However, reported outcomes differ depending on donor concentration, duration of exposure, and crop genotype. Thus, while NO participates in salinity tolerance networks, its effects are conditional and integrated with broader redox and hormonal regulation [49,62].

### 5.3. Temperature

Extreme temperatures disrupt membrane stability, enzymatic activity, and cellular metabolism, largely through ROS overproduction [63]. Under both heat and cold stress, altered NO accumulation has been observed, often preceding transcriptional and antioxidant adjustments.

During heat stress, NO has been linked to enhanced expression of heat shock proteins and modulation of antioxidant defenses. Some studies suggest that NO-dependent S-nitrosylation may affect protein stability or chaperone activity, although mechanistic resolution remains incomplete. Importantly, thermotolerance appears to depend on coordinated NO–ROS balance rather than isolated NO signaling [50].

Under cold stress, NO has been associated with membrane lipid remodeling and activation of cold-responsive TFs. Interaction with ROS and phytohormones likely contributes to these responses, but the relative contribution of NO varies among species and experimental systems [16,49]. As with other stresses, excessive NO accumulation under prolonged temperature extremes may shift from signaling to nitrosative injury, highlighting the importance of dose and timing.

### 5.4. Heavy Metal Stress

HM stress induces oxidative damage through enhanced ROS generation and interference with cellular metabolism [64,65,66]. Altered NO production under HM exposure has been documented in several plant species, where it appears to participate in redox regulation and detoxification processes [67].

Proposed mechanisms include NO-mediated enhancement of antioxidant enzyme activity and stimulation of metal-chelating molecules such as phytochelatins. Additionally, NO has been linked to transcriptional regulation of genes involved in metal transport, sequestration, and vacuolar compartmentalization [63,65,68]. These responses likely involve NO–ROS interplay and redox-sensitive TFs rather than direct NO action alone.

However, the protective versus deleterious role of NO under HM stress depends strongly on metal type, concentration, and duration of exposure. High or sustained NO levels may contribute to peroxynitrite formation and exacerbate cellular injury [69,70]. Therefore, NO-associated HM tolerance should be interpreted as a context-specific component of broader redox adaptation networks.

Across drought, salinity, temperature extremes, and heavy metal exposure, available evidence indicates that NO functions as a modulator within complex signaling networks integrating redox dynamics, hormonal pathways, ion transport, and transcriptional control. Rather than acting as a master regulator, NO appears to contribute to stress acclimation when produced at appropriate concentrations, in specific cellular compartments, and within a balanced ROS environment.

While mechanistic principles are broadly conserved across plant taxa, their translational relevance to horticultural crops lies in the sensitivity of these crops to environmental fluctuations and the economic importance of maintaining yield and quality under stress. Consequently, understanding the conditional and network-dependent role of NO provides a foundation for evaluating its potential—yet also its limitations—in sustainable stress management strategies.

## 6. NO-Mediated Stress Tolerance in Horticultural Crops

Horticultural crops, characterized by high economic value and quality-sensitive produce, are particularly vulnerable to abiotic stresses due to intensive cultivation systems and the narrow environmental thresholds required for optimal yield and marketability [38,58]. Accumulating evidence indicates that NO plays an important regulatory role in enhancing stress tolerance across diverse horticultural species [3]. However, unlike model plants, horticultural crops exhibit pronounced species-, organ-, and developmental stage-specific NO responses, reflecting differences in physiology, life cycle duration, and crop management practices [71]. This section synthesizes crop-specific NO-mediated stress responses, evaluates their implications for yield quality stabilization, and critically examines translational variability across horticultural systems. A summary of NO-mediated stress tolerance responses and associated yield and quality outcomes in major horticultural crops is provided in Table 1.

### 6.1. Crop-Specific Mechanistic Responses to NO Under Abiotic Stress

Evidence across vegetable, fruit, ornamental, and plantation crops suggests that NO frequently enhances tolerance to drought, salinity, temperature extremes, heavy metals, and nutrient imbalances. Nevertheless, the magnitude, persistence, and mechanistic basis of these responses differ substantially among crop categories and developmental stages [38,63].

In vegetable crops, including tomatoes, cucumbers, peppers, lettuce, spinach, and Pak choi, NO-mediated stress tolerance is primarily associated with rapid physiological adjustments. These include enhanced antioxidant enzyme activities, modulation of stomatal conductance, maintenance of photosynthetic efficiency, and improved osmotic adjustment under drought and salinity stress [63]. In salt-sensitive vegetables, regulation of ion transporter activity and maintenance of Na^+^/K^+^ homeostasis are particularly critical, as ionic imbalance directly constrains biomass accumulation and yield formation. Given their short life cycles, vegetable crops tend to rely on immediate redox-based protection and photosynthetic stabilization mechanisms.

In fruit crops, such as citrus, apples, grapevine, and strawberry, NO performs a dual role in stress mitigation and quality regulation. Under drought, salinity, iron deficiency, and temperature stress, NO enhances antioxidant defenses, stabilizes cellular membranes, and modulates hormone balance, thereby reducing stress-induced fruit drop and growth inhibition [49]. In perennial fruit trees, NO-mediated responses extend beyond acute stress protection to longer-term acclimation processes, including regulation of vascular function, carbohydrate partitioning, and source–sink relationships. These mechanisms are particularly relevant under prolonged or recurrent stress conditions associated with climate variability.

Ornamental crops, including chrysanthemum, roses, and petunias, exhibit NO-mediated improvements that are closely linked to aesthetic and post-harvest traits. NO delays stress-induced senescence, preserves pigment stability, and maintains membrane integrity, thereby sustaining flower color, size, and vase life under abiotic stress [50,72,73]. Because commercial value in ornamentals is tightly coupled to visual quality, even modest NO-induced improvements in membrane stability and oxidative protection can translate into substantial market benefits.

Comparative analysis across crop categories suggests that short-cycle vegetables predominantly benefit from rapid antioxidant and stomatal regulation mechanisms, whereas perennial fruit and plantation crops exhibit more integrated responses involving vascular regulation, carbohydrate allocation, and secondary metabolism. Such divergence reflects inherent differences in life history strategy, stress exposure duration, and developmental constraints. These distinctions underscore that NO-mediated stress tolerance cannot be uniformly generalized across horticultural systems.

### 6.2. Yield and Quality Stabilization Under NO-Mediated Stress Tolerance

Beyond stress survival, the agronomic significance of NO lies in its potential to stabilize yield and preserve produce quality under adverse environmental conditions [74]. Across multiple horticultural crops, NO supplementation or enhanced endogenous NO production has been associated with improvements in fruit set, fruit size, biomass accumulation, chlorophyll retention, and harvest index under stress.

These outcomes are largely attributable to NO-mediated protection of photosynthetic machinery, maintenance of water-use efficiency, stabilization of nutrient uptake, and mitigation of oxidative damage. By preserving source capacity and reducing premature senescence, NO can indirectly support reproductive development and yield continuity during stress episodes.

Importantly, NO also influences quality attributes that determine market value. In fruits and vegetables, NO has been associated with enhanced soluble sugar accumulation, improved sugar–acid balance, maintenance of vitamin content, and modulation of phenolic and flavonoid biosynthesis [57]. These effects contribute to improved antioxidant capacity and post-harvest stability. In ornamentals, NO-mediated delay of senescence and suppression of stress-induced tissue damage extend vase life and preserve commercial appearance [75,76].

However, these benefits are strongly dependent on concentration, timing, developmental stage, and mode of application. Excessive or prolonged NO exposure may disrupt cellular redox balance, promote nitrosative stress, or interfere with hormone signaling networks. Furthermore, many reported yield and quality improvements derive from controlled-environment or short-term studies, and their persistence under field conditions may vary. Therefore, while NO-mediated responses show considerable promise for buffering yield and quality losses, their practical effectiveness requires careful optimization and validation in crop- and context-specific scenarios.

### 6.3. Variability, Dose Dependency, and Translational Considerations

Despite consistent experimental evidence supporting NO involvement in horticultural stress tolerance, substantial variability exists among species, cultivars, and environmental contexts. Responses are often biphasic, with low to moderate NO levels promoting stress tolerance and higher concentrations exerting neutral or inhibitory effects. This concentration-dependent behavior highlights the importance of precise dosage management.

Additionally, different NO donors (e.g., sodium nitroprusside, S-nitrosoglutathione, and gaseous NO) vary in release kinetics, stability, and potential side effects, which may influence physiological outcomes and reproducibility. Environmental factors such as light intensity, temperature, soil properties, and irrigation regimes further modulate NO signaling and effectiveness.

A significant proportion of available data originates from laboratory or greenhouse experiments, whereas large-scale field validation remains comparatively limited. Variability in application methods, frequency, and crop growth stage at treatment complicates direct comparison among studies. These factors emphasize that NO-mediated stress tolerance should be interpreted as a context-dependent regulatory phenomenon rather than a universally transferable solution.

Collectively, current evidence supports a functional role for NO in enhancing stress resilience and stabilizing yield and quality in horticultural crops. However, effective translation into commercial systems requires integration of mechanistic understanding with optimized application strategies, field-level validation, and economic feasibility assessment.

### 6.4. Translational Interface: Linking NO Signaling to Agronomic Traits

While Section 3, Section 4 and Section 5 describe molecular and physiological mechanisms underlying NO-mediated stress responses, the translation of these processes into measurable agronomic outcomes requires integrative consideration. Improvements in antioxidant capacity, ion homeostasis, transcriptional reprogramming, and hormone modulation ultimately influence whole-plant performance, including biomass accumulation, reproductive development, fruit set, and post-harvest stability.

However, the quantitative contribution of specific NO-dependent signaling events to final yield or quality traits remains difficult to isolate. Stress intensity, genotype-specific responsiveness, environmental variability, and developmental stage all modulate the extent to which molecular signaling outcomes manifest at the crop level. In horticultural systems, where economic value depends heavily on produce quality parameters, small physiological adjustments may translate into substantial commercial effects.

Understanding this mechanistic-to-agronomic continuum is essential for evaluating the practical potential of NO-based strategies. Rather than assuming linear translation from signaling activation to yield improvement, a systems-level perspective is required to assess how NO-dependent processes interact with broader metabolic and environmental constraints.

**Table 1:** Nitric oxide-mediated stress tolerance responses and yield/quality outcomes in major horticultural crops.

Crop Category	Representative Crops	Major Abiotic Stresses	Key NO-Mediated Responses	Yield and Quality Outcomes	References
Vegetables	Tomatoes, cucumbers, peppers, and lettuce	Drought, salinity, and heat stress	Enhanced antioxidant activity, stomatal regulation, Na^+^/K^+^ homeostasis, and improved photosynthesis	Increased biomass and fruit yield; improved firmness, sugar content, and nutritional quality	[77,78,79,80]
Fruit crops	Citrus, apples, grapevine, and strawberries	Drought, salinity, Iron deficiency, and temperature extremes	Membrane stabilization, ROS scavenging, hormone-level balance, and vascular protection	Improved fruit set and size; reduced fruit drop; enhanced sugar–acid balance and antioxidant contents	[81,82,83,84]
Ornamental plants	Roses, chrysanthemums, and petunias	Heat, drought, and salinity	Delayed senescence, pigment stabilization, and improved membrane integrity	Maintained flower color and size; extended vase life and marketability	[85,86,87]
Plantation and spice crops	Tea, peppers	Chilling, heavy metals, and salinity	Redox-based regulation, secondary metabolite modulation, and enhanced stress acclimation	Stabilized yield; improved the quality of bioactive compounds	[88,89,90,91]
Leafy horticultural crops	Spinach, Pak choi	Salinity, nitrate, and flooding	Photosynthetic protection, osmotic adjustment, and antioxidant system enhancement	Improved leaf biomass, chlorophyll retention, and nutritional value	[92,93,94,95]

## 7. NO-Based Strategies for Sustainable Abiotic Stress Management

The regulatory role of NO in stress signaling has stimulated interest in its strategic use to enhance abiotic stress resilience while supporting sustainable horticultural production systems [96]. As a redox-active signaling molecule, NO can be exploited through priming, exogenous donor application, and integration with biostimulants or precision agriculture tools. However, effective translation from controlled experimental systems to open-field conditions requires careful consideration of concentration thresholds, release kinetics, delivery systems, crop specificity, and environmental variability. An overview of major NO-based strategies, their modes of application, benefits, and associated limitations is summarized in Table 2. 

### 7.1. Priming and Donor-Based Approaches

NO priming involves exposing seeds, seedlings, or plants to low, non-phytotoxic concentrations of NO or NO-releasing compounds to induce a physiological “stress memory,” enabling faster and more coordinated responses upon subsequent stress exposure [13]. Commonly used NO donors in experimental studies include sodium nitroprusside (SNP), S-nitrosoglutathione (GSNO), and diethylamine NONOate, each differing in release dynamics and stability.

A structured comparison of these donors highlights important translational considerations. SNP, although widely employed due to its rapid and reliable NO release, presents limitations associated with cyanide by-products and photoinstability under field conditions. GSNO, a more physiologically relevant donor, provides comparatively controlled NO release and participates in endogenous S-nitrosylation cycles, yet its chemical stability and production cost may restrict large-scale application. NONOates offer tunable release kinetics depending on their chemical structure; however, their decomposition rates are strongly influenced by temperature and pH, which may reduce predictability under open-field conditions. Emerging controlled-release and nano-encapsulation systems aim to enhance NO stability, minimize volatilization, and improve delivery efficiency, although these approaches remain largely at experimental or pilot stages. Collectively, these differences indicate that donor selection must consider not only biological efficacy but also environmental stability, safety profile, regulatory acceptance, and economic feasibility.

NO priming has been associated with enhanced antioxidant capacity, improved osmotic adjustment, stabilization of photosynthetic machinery, and modulation of hormone-responsive pathways under drought, salinity, temperature extremes, and heavy metal stress [51]. In horticultural crops, seed priming often improves germination rate, seedling vigor, and early establishment under suboptimal conditions, potentially conferring advantages that persist into later developmental stages.

Nevertheless, priming responses are strongly concentration-dependent and may vary among species and cultivars. The biphasic nature of NO signaling implies that suboptimal concentrations may be ineffective, whereas excessive doses can induce nitrosative stress or disrupt endogenous hormonal balance. Moreover, the persistence of priming-induced benefits under prolonged field stress remains insufficiently characterized, highlighting the need for multi-season validation studies.

Exogenous donor application through foliar spray or root drenching provides more immediate stress mitigation. However, donor-specific properties—including NO release rate, photostability, interaction with soil components, and potential by-product toxicity—significantly influence efficacy. For instance, while SNP is widely used in laboratory research, concerns regarding cyanide release and environmental accumulation may limit its large-scale agricultural applicability. Therefore, identification and development of safer, controlled-release NO formulations remain critical for field-level deployment.

### 7.2. Integration of NO with Biostimulants and Eco-Friendly Management Practices

The integration of NO-based approaches with biostimulants represents a promising avenue for sustainable stress management. Biostimulants such as seaweed extracts, humic substances, amino acids, and plant growth-promoting microorganisms may enhance endogenous NO production or amplify NO-dependent signaling cascades, thereby reinforcing stress adaptation mechanisms [97,98].

Microbial inoculants that improve root architecture and nutrient acquisition can synergize with NO-mediated regulation of water-use efficiency and ionic balance under drought and salinity stress. Similarly, combined application of NO donors with organic amendments or natural extracts has been associated with enhanced antioxidant defense and metabolic flexibility, potentially reducing dependence on synthetic agrochemicals.

However, synergistic interactions are formulation- and crop-specific. Variability in microbial activity, soil chemistry, and environmental conditions can alter NO bioavailability and signaling outcomes. Standardization of combined treatments and evaluation of compatibility with organic and integrated production systems are necessary before recommending widespread adoption.

### 7.3. Role of NO in Climate-Resilient Horticultural Systems

Climate change is intensifying the frequency and severity of abiotic stresses, including drought, heat waves, salinity intrusion, and soil contamination. NO is implicated in rapid stress perception, redox-based signal integration, and hormonal crosstalk, processes that collectively enable plants to respond to complex and simultaneous stress scenarios [3,99].

NO-mediated cross-tolerance—where exposure to one stress enhances resistance to others—has been reported in several horticultural species. This capacity may be particularly valuable under unpredictable climatic fluctuations. By stabilizing photosynthesis, mitigating oxidative damage, and preserving reproductive development, NO-associated responses may contribute to yield and quality stability under stress.

However, the role of NO in climate resilience should be interpreted cautiously. Most available evidence derives from controlled stress simulations, whereas real-world stress combinations involve fluctuating intensity, duration, and environmental interactions. Long-term, field-based assessments across diverse agroecological zones are required to validate the consistency and economic viability of NO-based interventions under climate-smart agriculture frameworks.

### 7.4. Practical Constraints and Field-Level Challenges

Despite encouraging experimental findings, several limitations constrain the large-scale adoption of NO-based strategies [15,16]. The intrinsic reactivity and short half-life of NO complicate controlled delivery and sustained efficacy in open-field conditions. Environmental factors such as light intensity, temperature, soil pH, microbial activity, and irrigation regimes can alter NO stability and plant responsiveness [49,100].

Donor-related concerns remain a critical issue. Some commonly used NO donors may generate toxic by-products or accumulate in soil systems if mismanaged. Development of biodegradable, slow-release, and environmentally safe NO formulations is therefore a priority. Additionally, crop-specific sensitivity and developmental stage-dependent responses hinder the establishment of universal application protocols.

Economic considerations—including production cost, scalability, compatibility with existing agronomic practices, and regulatory approval—must also be addressed. Without clear cost–benefit advantages and standardized guidelines, adoption by growers may remain limited.

Consequently, advancing NO-based stress management requires interdisciplinary collaboration among plant physiologists, agronomists, formulation chemists, and regulatory experts. Integrating mechanistic insights with field validation, precision delivery technologies, and economic assessment will determine whether NO-based strategies can transition from experimental promise to practical agricultural solutions.

**Table 2:** NO-based strategies for sustainable abiotic stress management: benefits and limitations.

Strategy	Mode of Application	Target Stresses	Key Benefits	Major Limitations	References
NO priming	Seed soaking, seedling treatment, and foliar spray	Drought, salinity, temperature extremes, and heavy metals	Induces stress memory; enhances antioxidant capacity; low input requirement	Dosage sensitivity; limited persistence under field conditions	[12,19,101,102,103]
NO donor application	Foliar spray and root drench	Salinity, heavy metals, and heat	Rapid stress mitigation improves	Donor toxicity risks; short NO half-life	[67,104,105]
NO–biostimulant integration	Combined application with organic extracts or microbes	Multiple abiotic stresses	Synergistic effects; eco-friendly; improves nutrient uptake and stress tolerance	Variable efficacy which depends on the formulation and crop type	[3,6,8,106]
NO-mediated climate resilience	Integrated into climate-smart management	Combined and recurring stresses	Enhances cross-tolerance and yield stability under climate variability	Requires long-term field validation	[36,107]
Precision NO delivery systems	Controlled-release formulations and targeted sprays	Stress-prone environments	Improved efficiency and reduced environmental risk	High development costs; limited commercial availability	[106,108]

## 8. Knowledge Gaps and Future Perspectives

Despite substantial progress in elucidating NO-mediated mechanisms of abiotic stress tolerance, several critical knowledge gaps constrain the translation of experimental findings into sustainable horticultural practice. A large proportion of current evidence derives from controlled laboratory or greenhouse studies, where stress conditions are simplified and temporally uniform. In contrast, field environments involve fluctuating stress intensities, interacting abiotic and biotic factors, and heterogeneous soil–plant–microbial dynamics. Under such conditions, the highly reactive and transient nature of NO presents significant challenges for controlled delivery, sustained bioavailability, and reproducible physiological outcomes. Long-term, multi-location field trials are therefore essential to evaluate the agronomic reliability, economic feasibility, and environmental safety of NO-based interventions.

Another major challenge concerns the pronounced crop specificity and context dependency of NO signaling. Although core redox and hormonal interactions are broadly conserved, downstream physiological and molecular responses vary widely among species, cultivars, developmental stages, and stress combinations [109]. The biphasic and concentration-dependent behavior of NO further complicates practical application, as suboptimal levels may be ineffective whereas excessive accumulation can induce nitrosative stress or disrupt endogenous regulatory networks. For many horticultural crops, optimal dosage ranges, timing strategies, and donor selection criteria remain insufficiently defined. Differences between annual and perennial species, as well as between vegetative and reproductive growth phases, necessitate crop- and stage-specific optimization rather than generalized protocols.

Future progress will depend on integrating NO biology with advances in molecular breeding, omics technologies, and precision agriculture. High-throughput transcriptomic, proteomic, and metabolomic approaches provide opportunities to identify NO-responsive regulatory hubs, stress-associated pathways, and candidate markers linked to improved tolerance. Such insights may facilitate the development of genotypes with enhanced endogenous NO regulation or greater responsiveness to exogenous NO treatments. However, incorporation of NO-related traits into breeding programs will require robust phenotyping frameworks and validation under realistic environmental conditions.

In parallel, precision agriculture technologies—including real-time stress monitoring, sensor-based diagnostics, and targeted delivery systems—may improve the spatial and temporal accuracy of NO applications. Coupling mechanistic understanding with data-driven decision support platforms could enhance efficacy while minimizing environmental risks. Nevertheless, technological feasibility, cost-effectiveness, and regulatory considerations must be evaluated alongside biological performance.

Overall, advancing NO-based stress management strategies requires interdisciplinary collaboration that bridges molecular signaling research with agronomy, formulation science, crop improvement, and digital agriculture. Only through such integrative efforts can mechanistic insights be translated into reliable and context-appropriate tools for sustainable horticultural production.

## 9. Conclusions

NO is widely recognized as a versatile signaling molecule involved in plant responses to abiotic stress. By integrating redox regulation, hormonal crosstalk, ion homeostasis, and transcriptional control, NO contributes to coordinated adjustment of physiological and metabolic processes under adverse environmental conditions. Evidence across multiple stress types—including drought, salinity, temperature extremes, and heavy metal toxicity—indicates that NO functions not as an isolated regulator but as a dynamic component of broader stress-responsive networks.

In horticultural crops, NO-associated signaling has been linked to improved stress tolerance and, in several cases, stabilization of yield- and quality-related traits. These effects highlight its potential agronomic relevance, particularly in high-value systems where environmental fluctuations strongly influence productivity and market quality. Strategies such as priming, donor application, and integration with biostimulants offer promising experimental avenues for enhancing resilience. However, the effectiveness of these approaches remains highly dependent on species, developmental stage, stress context, and application parameters.

Importantly, current knowledge is still weighted toward controlled-environment research, and comprehensive field validation remains limited. Practical deployment of NO-based strategies will require precise dosage optimization, environmentally safe delivery systems, regulatory evaluation, and clear cost–benefit justification. Without such considerations, the transition from experimental success to commercial implementation may remain constrained.

Future advances should prioritize multi-season field trials, crop-specific optimization frameworks, and integration with breeding and precision agriculture technologies. By aligning mechanistic insight with agronomic validation and sustainability principles, NO-centered approaches may contribute meaningfully—though not exclusively—to the development of climate-resilient and resource-efficient horticultural production systems.

## Data Availability

The original contributions presented in this study are included in the article. Further inquiries can be directed to the corresponding authors.
